# Stopwords in technical language processing

**DOI:** 10.1371/journal.pone.0254937

**Published:** 2021-08-05

**Authors:** Serhad Sarica, Jianxi Luo

**Affiliations:** 1 Institute of High Performance Computing, Agency for Science, Technology and Research, Singapore, Singapore; 2 Data-Driven Innovation Lab, Singapore University of Technology and Design, Singapore, Singapore; University of Sao Paulo, BRAZIL

## Abstract

There are increasing applications of natural language processing techniques for information retrieval, indexing, topic modelling and text classification in engineering contexts. A standard component of such tasks is the removal of stopwords, which are uninformative components of the data. While researchers use readily available stopwords lists that are derived from non-technical resources, the technical jargon of engineering fields contains their own highly frequent and uninformative words and there exists no standard stopwords list for technical language processing applications. Here we address this gap by rigorously identifying generic, insignificant, uninformative stopwords in engineering texts beyond the stopwords in general texts, based on the synthesis of alternative statistical measures such as term frequency, inverse document frequency, and entropy, and curating a stopwords dataset ready for technical language processing applications.

## 1. Introduction

Natural language processing (NLP) and text analysis have been growingly popular in engineering analytics [1–6]. To ensure the accuracy and efficiency of such NLP tasks as indexing, topic modelling, text classification and information retrieval [7–11], the uninformative words, often referred to as “stopwords”, need to be removed in the pre-processing step. Stopwords frequently appear in many different natural language documents or parts of the text in a document but carry little information about the part of the text they belong to. Hence, the removal of stopwords can increase the signal-to-noise ratio in unstructured text and thus increase the statistical significance of terms that may be important for a specific task. Example stopwords include”each”,”about”,”such”, and”the”.

There have been efforts to identify stopwords from generic knowledge sources such as Brown Corpus [10, 12], 20 newsgroup corpus [8], books corpus [13], etc, and curate a generic stopwords list for removal in NLP applications across fields. The use of such a standard stopwords list, e.g. the one distributed with the popular Natural Language Tool Kit (NLTK) [14] python package, for removal in data pre-processing has become an NLP standard in both research and industry.

These standard stopwords lists are also utilized in the text pre-processing steps of many engineering design studies focusing on tasks such as topic modelling [15–17], feature extraction [18, 19], design information extraction [20, 21], design representation [22–25], text classification [26], semantic network and ontology construction [4, 27–29] and query completion [20, 30].

However, the technical language used in engineering or technical texts is different from layman languages and may use stopwords that are less prevalent in layperson languages. When it comes to engineering or technical text analysis, researchers and engineers either just adopt the readily available generic stopwords lists for removal [1–4], leaving many domain-related uninformative and repetitive terms in the data or identify additional stopwords in a manual, ad hoc or heuristic manner [7, 31–33]. There exist no standard stopwords list for technical language processing applications.

Here, we address this gap by rigorously identifying generic, insignificant, uninformative stopwords in engineering texts beyond the stopwords in general texts, based on the synthesis of alternative statistical measures such as term frequency, inverse document frequency and entropy. The resultant stopwords dataset is statistically identified and human-evaluated. Researchers, analysts, and engineers working on technology-related textual data and technical language analysis can directly apply it to denoise and filter their technical textual data without conducting the manual and ad hoc discovery and removal of uninformative words by themselves. We exemplified such a use case to measure the effectiveness of our new stopwords dataset in text classification tasks.

## 2. Proposed approach

To identify stopwords in technical language texts, we statistically analyze the natural texts in patent documents which are descriptions of technologies at all levels. The patent database is vast and provides the most comprehensive coverage of technological domains. Specifically, our patent text corpus contains 687,442,479 tokens (words, bi-, tri- and four-grams) from 31,567,141 sentences of the titles and abstracts of 6,824,356 of utility patents in the complete USPTO patent database from 1976 to 29^th^ September 2020 (access date: 5 January 2021). Non-technical design patents are excluded. Technical description fields are avoided because they include information on contexts, backgrounds, and prior arts that may be non-relevant to the specific invention and repetitive, lead to statistical bias and increase computational requirements. We also avoided legal claim sections that are written in redundant, disguising, and legal terms.

In text analysis for topic modelling, text classification or information retrieval, various statistical metrics, such as term frequency (TF) [9, 11], inverse-document frequency (IDF) [9], term-frequency-inverse-document-frequency (TFIDF) [7], entropy [13, 34], information content [34], information gain [35] and Kullback-Leibler divergence [9], are employed to sort the words in a corpus [8, 35]. Herein we use TF, TFIDF, and information entropy to automatically identify candidate stopwords.

Furthermore, some of the technically significant terms such as “composite wall”, “driving motion”, and “hose adapter” are statistically indistinguishable from such stopwords “be”, “and” and “for”, regardless of the statistic metrics for sorting. That is, automatic and data-driven methods by themselves are not accurate and reliable enough to return stopwords. Therefore, we also use a human-reliant step to further evaluate the automatically identified candidate stopwords and confirm a final set of stopwords that do not carry information on engineering and technology.

**Fig 1:**
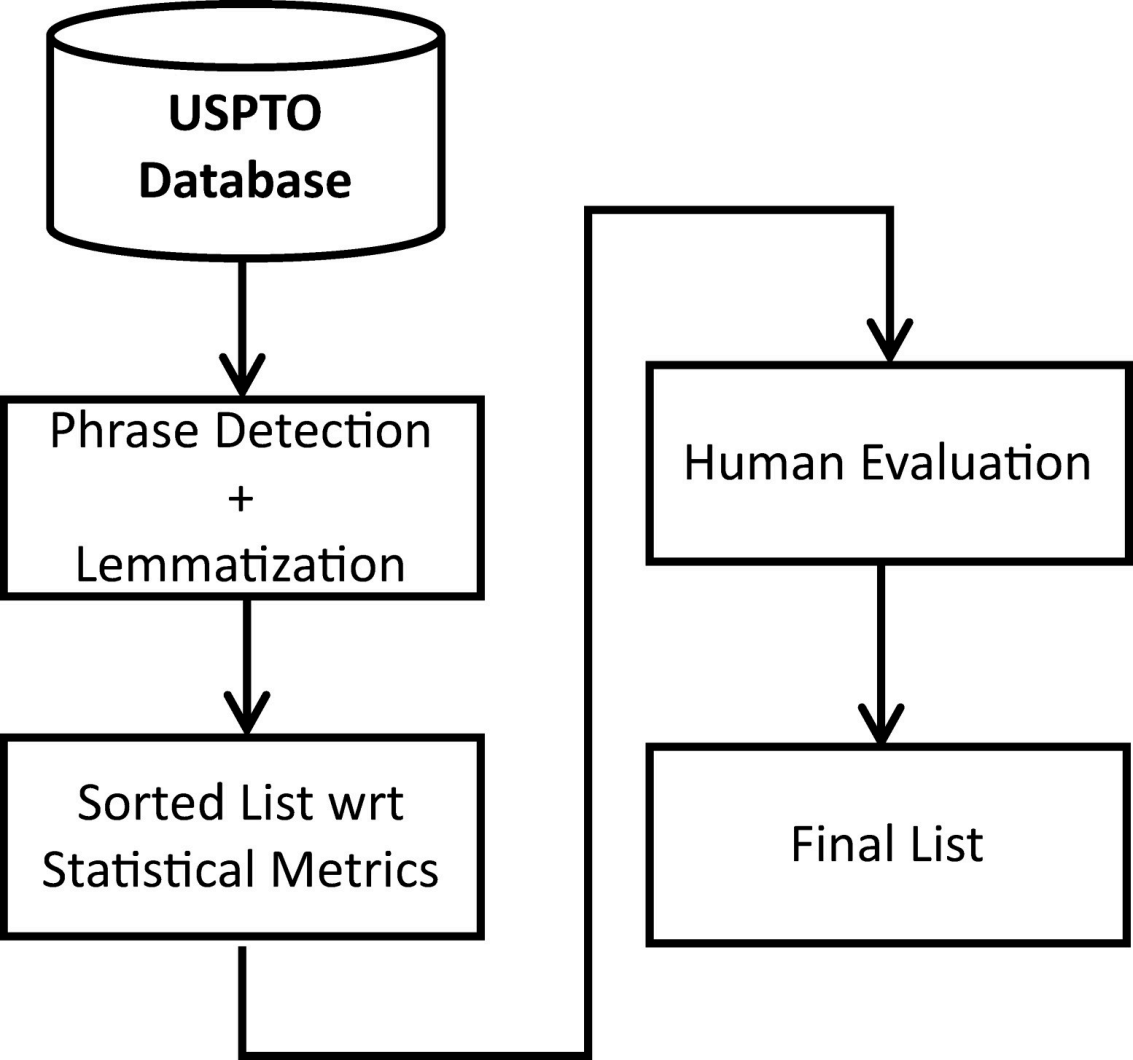
Overall procedure.

In brief, the overall procedure as depicted in Fig 1 consists of three major steps: 1) basic pre-processing of the patent natural texts, including punctuation removal, lowercasing, phrase detection, and lemmatization; 2) using multiple statistic metrics from NLP and information theory to identify a ranked list of candidate stopwords; 3) term-by-term evaluation by human experts on their insignificancy for technical texts to confirm stopwords that are uninformative about engineering and technology. In the following, we describe the implementation details of these three steps.

## 3. Implementation

### 3.1. Pre-processing

The patent texts in the corpus are first transformed into a line-sentence format, utilizing the sentence tokenization method in the NLTK, and normalized to lowercase letters to avoid additional vocabulary caused by lowercase/uppercase differences of the same words. The original raw texts are transformed into a collection of 31,567,141 sentences, including 829,843,528 unigrams.

Phrases are detected with the algorithm of Mikolov et al. [36] that finds words that frequently appear together, and in other contexts infrequently, by using a simple statistical method based on the count of words to give a score to each bigram such that:

$$score(w_{i},w_{j})=\frac{\left(count(w_{i}w_{j})-\delta )|N\right|}{count(w_{i})count(w_{j})},$$ (1)

where *w*_*i*_ is a term, *count(w*_*i*_*w*_*j*_*)* is the count of *w*_*i*_ and *w*_*j*_ appearing together as bigrams in the collection of sentences and *count(w*_*i*_*)* is the count of *w*_*i*_ in the collection of sentences. *δ* is the discounting coefficient to prevent too many phrases consisting of very infrequent words, and set *δ = 1* to avoid having scores higher than 0 for phrases occurring less than twice. The term *N* = ∑_*t,p*∈*P*_*n*(*t,p*) represents the total number of tokens in the patent database. Bigrams with a score over a defined threshold (*T*_*phrase*_) are considered as phrases and joined with a “_” character in the corpus, to be treated as a single term. We run the phrasing algorithm of Mikolov et al. (2013) on the pre-processed corpus twice to detect *n*-grams, where *n* = [2, 4]. The first run detects only bigrams by employing a higher *T*_*phrase*_ value, while the second run can detect *n*-grams up to *n* = 4 by using a lower *T*_*phrase*_ value to enable combinations of bigrams. Via this procedure of repeating the phrase detection process with decreasing threshold values of *T*_*phrase*_, we identified phrases that appear more frequently in the first step using the higher threshold value, e.g., “autonomous vehicle”, and discovered phrases that are comparatively less frequent in the second step using the lower threshold value, e.g., “autonomous vehicle platooning”. In this study, we used the best performing thresholds (5, 2.5) found in a previous study [31].

The phrase detection computation resulted in a vocabulary of 7,309,577 terms, including 5,459,998 phrases. Since the adopted phrase detection algorithm is purely based on co-occurrence statistics, the detection of some faulty phrases including stopwords such as “the_”, “a_”, “and_”, and “to_” or including punctuation marks is inevitable. Therefore, the detected phrases are processed one more time to split the known stopwords from the NLTK [14] and USPTO [37] stopwords lists and punctuation marks. For example, “an_internal_combustion_engine” is replaced with “an internal_combustion_engine”. Then the vocabulary is reduced to 3,461,271 terms, including 1,818,836 phrases.

Next, all the words are represented with their regularized forms to avoid having multiple terms representing the same word or phrase. This step is achieved by first using a POS tagger [38] to detect the type of words in the sentences and lemmatize those words accordingly. For example, if the word “learning” is tagged as a VERB, it would be regularized as “learn” while it would be regularized as “learning” if it is tagged as a NOUN. The lemmatization procedure further decreased the vocabulary to 3,259,037 terms, including 1,632,239 phrases.

As the last step, we removed the words in the famous NLTK [14] and USPTO [37] stopwords lists. The NLTK stopwords list focuses more on general stopwords that can be encountered in daily English language such as “a, an, the, …, he, she, his, her, …, what, which, who, …”, in total 179 words. On the other hand, the USPTO stopwords list includes words that occur very frequently in patent documents and do not contain critical meaning within patent texts, such as “claim, comprise, … embodiment, … provide, respectively, therefore, thereby, thereof, thereto, …”, in total 99 words. The union of these two lists contains 220 stopwords.

**Fig 2:**
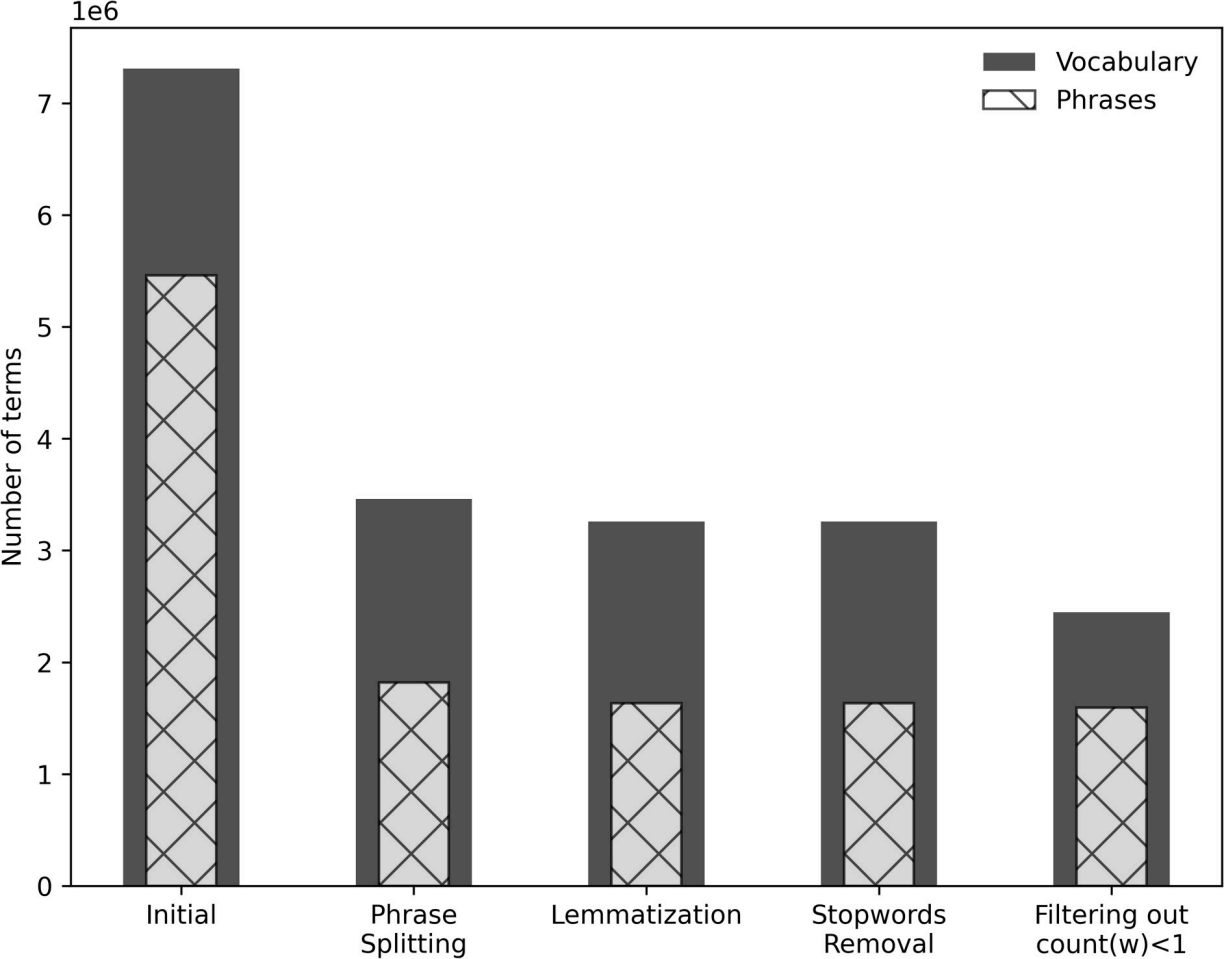
The reduction in the size of the vocabulary through pre-processing steps.

Additionally, we also discarded the words appearing only 1 time in the whole patent database, which leads to a final set of 2,448,125 terms, including 1,594,073 phrases. The reduction in the size of the vocabulary through pre-processing steps is presented in Fig 2.

### 3.2. Term statistics

To identify the frequently occurring words or phrases that carry little information content about engineering and technology, we use four metrics together: 1) direct term frequency (TF), 2) inverse-document frequency (IDF), 3) term-frequency-inverse-document-frequency (TFIDF), and 4) Shannon’s information entropy [39].

Consider a corpus *C* of patents. We use *TF(t)* to denote the frequency of term *t* with the equation:

TF(t)=n(t)/N, (2)

where *n*(*t*) = ∑_*p*∈*P*_*n*(*t,p*) is the total count of the term *t* in all patents, and *N* = ∑_*t,p*∈*C*_*n*(*t,p*) is the total number of terms in all patents where *N* is constant for a given *C*. The term frequency is an important indicator of the commonality of a term within a collection of documents. Stopwords are expected to have high term frequency.

Inverse-document-frequency (IDF) is calculated as follows:

$$IDF(t)=\log\frac{\left|C\right|}{DF(t)}$$ (3)

where *DF*(*t*) = |{*pϵC*: *tϵp*}| is the number of patents containing term *t* and |*C*| represents the number of patents in the database. This metric penalizes the frequently occurring terms and favours the ones occurring in a few documents only. The metric’s lower bound is 0 which refers to the terms that appear in every single document in the database. The upper bound is defined by the terms appearing only in one document, which is log |*C*|.

Term frequency-inverse-document-frequency (TFIDF) is calculated as follows:

$$TFIDF(t)=\frac{1}{DF(t)}\sum_{p}\frac{n(t,p)}{n(p)}\frac{\left|C\right|}{DF(t)},$$ (4)

where *n*(*t,p*) is the count of the term *t* in the patent *p* and *n*(*p*) is the total number of terms in the patent *p*. This metric favours the terms that appear in a few documents, with a considerably high term frequency within the document. If a term appears in many documents, its TFIDF score will be penalized by the IDF score due to its commonality. Here, we did not use the traditional IDF metric but removed the log normalizing function to penalize the terms commonly occurring in the entire patent database harder, regardless of their in-document (patent) term frequencies. We eventually used the mean of the single document TFIDF scores for each term.

The entropy of the term *t* is calculated as follows. The metric indicates how uneven the distribution of term *t* is in corpus *C*.

$$H(t|C)=-\sum_{p}P(p|t)logP(p|t),$$ (5)

where *P*(*p|t*) = *n*(*t,p*)/*n*(*t*) is the distribution of the term *t* over patent documents. This indicates how evenly distributed a term is in the patent database. Maximum attainable entropy value for a given collection of documents is an even distribution to all patents which leads to log |*C*|. Therefore, the terms having higher entropy values will contain less information about the patents where they appear, compared to other terms with lower entropy.

**Fig 3:**
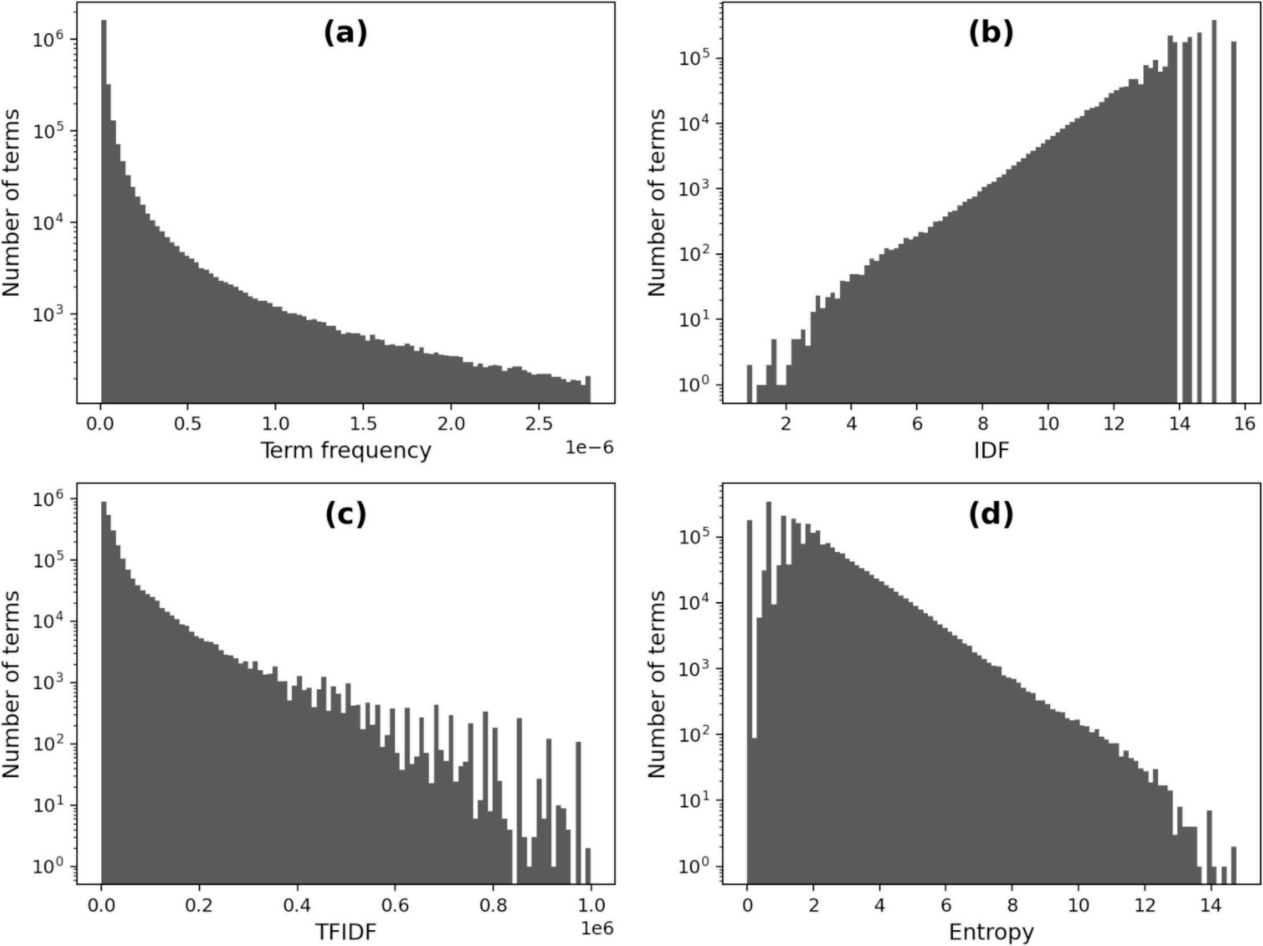
Distribution of terms by (a) Term frequency, (b) IDF, (c) TFIDF and (d) Entropy. Term frequency and tfidf histograms arbitrarily filtered (term-count< = 1000, TFIDF score< = 10^6^) for visualization purposes. In fact, they have longer right tails.

We reported the distributions of terms in our corpus according to these four metrics in Fig 3. The *term-frequency* distribution has a very long right tail, indicating most of the terms appear a few times in the patent database while some words appear so frequently. Our further tests found that the distribution follows the a power law [40, 41]. By contrast, the distribution by IDF has a long left-tail, indicating the existence of a few terms that appears commonly in all patents. The TFIDF distribution also has a long right tail that indicates the existence of highly common terms in each patent and highly strong domain-specific terms dominating a set of patents. Moreover, the long right-tail of entropy distribution indicates comparingly few high valued terms that are appearing commonly in the entire database. Therefore, assessing the four metrics together will allow us to detect the stopwords with varied occurrence patterns.

### 3.3. Human evaluation

As a first step, we formed four different lists of terms sorted by decreasing TF, increasing IDF, increasing TFIDF, and decreasing entropy. S1 Table presents the top-ranked 30 terms in respective lists, which differ but largely overlap. As the second step, the top 2,000 terms in each of the four lists are used to form a union set of terms. The union only includes 2,257 terms, which indicates that the lists based on four alternative statistic metrics overlap significantly. Then, the terms in the union set are evaluated by two researchers with more than 20 years of engineering experience each, in terms of whether a term carries information about engineering and technology, to identify stopwords. The researchers initially achieved an inter-rater reliability of 0.83 according to Cronbach’s alpha [42] and then discussed the discrepancies to reach a consensus on a final list of 62 insignificant terms.

### 3.4. Final list

This list, compared to our previous study, which identified a list of stopwords [31] (see S2 Table) by manually reading 1,000 randomly selected sentences from the same patent text corpus, includes 26 new uninformative stopwords that the previous list did not cover. In the meantime, we also found the previous list contains other 25 stopwords, which are still deemed qualified stopwords in this study. Therefore, we integrate these 25 stopwords from the previous study with the 62 stopwords identified here to derive a final list of 87 stopwords for technical language analysis. The final list is presented in Table 1 together with the NLTK stopwords list and the USPTO stopwords list. It is suggested to apply the three stopwords lists together in technical language processing applications across technical fields.

**Table 1:** Stopwords lists for technical language processing applications.

NLTK Stopwords List [14] (179 words)		USPTO Stopwords List [37] (99 words)		This Study (87 words)	
a	needn	a	not	able	never
about	needn’t	accordance	now	above-	often
above	no	according	of	mentioned	others
after	nor	all	on	accordingly	otherwise
again	not	also	onto	across	overall
against	now	an	or	along	rather
ain	o	and	other	already	remarkably
all	of	another	particularly	alternatively	significantly
am	off	are	preferably	always	simply
an	on	as	preferred	among	sometimes
and	once	at	present	and/or	specifically
any	only	be	provide	anything	straight
are	or	because	provided	anywhere	forward
aren	other	been	provides	better	substantially
aren’t	our	being	relatively	disclosure	thereafter
as	ours	by	respectively	due	therebetween
at	ourselves	claim	said	easily	therefor
be	out	comprises	should	easy	therefrom
because	over	corresponding	since	e.g	therein
been	own	could	some	either	thereinto
before	re	described	such	elsewhere	thereon
being	s	desired	suitable	enough	therethrough
below	same	do	than	especially	therewith
between	shan	does	that	essentially	together
both	shan’t	each	the	et al	toward
but	she	embodiment	their	etc	towards
by	she’s	fig	then	eventually	typical
can	should	figs	there	excellent	upon
couldn	should’ve	for	thereby	finally	via
couldn’t	shouldn	from	therefore	furthermore	vice versa
d	shouldn’t	further	thereof	good	whatever
did	so	generally	thereto	hence	whereas
didn	some	had	these	he/she	whereat
didn’t	such	has	they	him/her	wherever
do	t	have	this	his/her	whether
does	than	having	those	ie	whose
doesn	that	herein	thus	ii	within
doesn’t	that’ll	however	to	iii	without
doing	the	if	use	instead	yet
don	their	in	various	later	
don’t	theirs	into	was	like	
down	them	invention	were	little	
during	themselves	is	what	many	
each	there	it	when	may	
few	these	its	where	meanwhile	
for	they	means	whereby	might	
from	this		wherein	moreover	
further	those		which	much	
had	through		while	must	
hadn	to		who		
hadn’t	too		will		
has	under		with		
hasn	until		Would		
hasn’t	up				
have	ve				
haven	very				
haven’t	was				
having	wasn				
he	wasn’t				
her	we				
here	were				
hers	weren				
herself	weren’t				
him	what				
himself	when				
his	where				
how	which				
i	while				
if	who				
in	whom				
into	why				
is	will				
isn	with				
isn’t	won				
it	won’t				
it’s	wouldn				
its	wouldn’t				
itself	y				
just	you				
ll	you’d				
m	you’ll				
ma	you’re				
me	you’ve				
mightn	your				
mightn’t	yours				
more	yourself				
most	yourselves				
mustn					
mustn’t					
my					
myself					

## 4. Case study evaluation

Next, we use a case study to show the usefulness of the resultant stopwords list in a common NLP task, namely text classification.

### 4.1. Data

We use patent database as the data source in the subsequent case study. Every patent is classified according to the Cooperative Patent Classification (CPC) scheme. CPC is hierarchical, with 8 broad “Sections” (e.g., G: Physics) at the highest level. Each Section is comprised of Sub-Sections (e.g., G06: Computing; calculating; counting), which are further decomposed into Groups (e.g. G06F: Electric digital data processing).

We chose one CPC Group from each Section (Table 2) and randomly sample 10,000 patents (or all of the patents within the group, whichever is smaller) from each CPC Group to curate a dataset of patent documents in 8 distinct clusters. Because a CPC Group represents a highly specialized technology domain, the patent documents from the same CPC Group should share highly similar technical concepts in their texts. Also, because the 8 CPC Groups are drawn from 8 different broadly-defined sections, the patent documents from different sections describe very different technologies and are expected to contain semantically dissimilar terms. In so doing, we curate 8 distinct classes of documents, whose topics are highly similar within each class and highly dissimilar across classes. The dataset is further filtered by selecting the patents with at least one stopword from the NLTK+USPTO set and at least one stopword from the new list introduced in this study.

**Table 2:** Selected CPC groups for text classification tasks.

CPC Section	Definition	Selected CPC Group	Definition
A	Human necessities	A01K	Housing animals; equipment therefor
B	Performing operations; transporting	B01D	Separation
C	Chemistry; metallurgy	C06B	Explosives or thermic compositions; manufacture thereof; use of single substances as explosives
D	Textiles; paper	D21F	Paper-making machines; methods of producing paper thereon
E	Fixed constructions	E01H	Street cleaning; cleaning of permanent ways; cleaning beaches; dispersing {or preventing} fog in general {cleaning street or railway furniture or tunnel walls}
F	Mechanical engineering; lighting; heating; weapons; blasting engines or pumps	F02B	Internal-combustion piston engines; combustion engines in general
G	Physics	G06F	Electric digital data processing
H	Electricity	H04B	Transmission of information carrying signals

### 4.2. Text classification

We tested the usefulness of the proposed technical stopwords list in a text classification task by training and using a Long Short Term Memory (LSTM) [43] model on the dataset we acquired. In the experiment, the maximum length of every patent text is limited to 500 words, which are long enough to cover typical patent abstract and title texts. We employed an embedding layer of 300 neurons, followed by a dropout layer which is directly connected to an LSTM layer of 100 units. As the last layer, we employed a softmax classifier.

We trained three different models by using three different training sources: Patent titles and abstracts (1) with all the words and phrases, (2) without NLTK and USPTO stopwords, and (3) without NLTK, USPTO and technical stopwords we identified in this study. Furthermore, we randomly selected 100 patents from each CPC Group given in Table 2 to be used as the test set. The rest of the patent texts are used in the training.

Table 3 summarizes the results. We measured macro averaged precision, recall and accuracy metrics as the indicators for the different models’ performances in classifying the patent texts to the selected eight different CPC groups. In general, cleaning text by removing stopwords significantly increases the performance. Removing our new list of technical stopwords in addition to NLTK and USPTO stopwords in pre-processing further improves the prediction performance in this text classification task.

**Table 3:** Macro average of precision, recall and accuracy of multi-class text classification (LSTM) task predictions.

Stopwords Removal Scenarios	Precision	Recall	Accuracy
Vocabulary #1 (Raw texts)	0.887	0.849	0.849
Vocabulary #2 (NLTK, USPTO stopwords removed)	0.961	0.959	0.959
Vocabulary #3 (NLTK, USPTO, Technical stopwords removed)	0.971	0.970	0.970

## 5. Concluding remarks

To develop a comprehensive list of stopwords in engineering and technology-related texts, we mined the patent text database with several statistical metrics from term frequency to entropy together to automatically identify candidate stopwords and use human evaluation to validate, screen and finalize stopwords from the candidates. In this procedure, the automatic data-driven detections of four statistic metrics yield highly overlapping results, and the human evaluations also came with high inter-rater reliability, suggesting evaluator independence. Our final stopwords list can be used as a complementary list to NLTK and USPTO stopwords lists in NLP and text analysis tasks related to technology, engineering, and innovation.

## Supporting information

S1 Table Top 30 terms for term-frequency, IDF, TFIDF and entropy. (PDF)

S2 Table The stopwords identified in the previous study. * indicates that the term is also identified in the current study. + indicates that the term is a stopwords as defined in the current study. Rest of the terms are no longer considered as stopwords as defined in the current study.
(PDF)

S1 Nomenclature (DOCX)
