# Correction: Stopwords in technical language processing

**DOI:** 10.1371/journal.pone.0315195

**Published:** 2024-12-05

**Authors:** Serhad Sarica, Jianxi Luo

In Table 1, there is an error in column five rows two and three. The words above and mentioned should be in a single row. Please see the correct Table 1 here.

**Table 1:** Stopwords lists for technical language processing applications.

NLTK Stopwords List [14] (179 words)				USPTO Stopwords List [37] (99 words)		This Study (87 words)	
a	hadn’t	on	wasn’t	a	onto	able	others
about	has	once	we	accordance	or	above-mentioned	otherwise
above	hasn	only	were	according	other	accordingly	overall
after	hasn’t	or	weren	all	particularly	across	rather
again	have	other	weren’t	also	preferably	along	remarkably
against	haven	our	what	an	preferred	already	significantly
ain	haven’t	ours	when	and	present	alternatively	simply
all	having	ourselves	where	another	provide	always	sometimes
am	he	out	which	are	provided	among	specifically
an	her	over	while	as	provides	and/or	straight forward
and	here	own	who	at	relatively	anything	substantially
any	hers	re	whom	be	respectively	anywhere	thereafter
are	herself	s	why	because	said	better	therebetween
aren	him	same	will	been	should	disclosure	therefor
aren’t	himself	shan	with	being	since	due	therefrom
as	his	shan’t	won	by	some	easily	therein
at	how	she	won’t	claim	such	easy	thereinto
be	i	she’s	wouldn	comprises	suitable	e.g	thereon
because	if	should	wouldn’t	corresponding	than	either	therethrough
been	in	should’ve	y	could	that	elsewhere	therewith
before	into	shouldn	you	described	the	enough	together
being	is	shouldn’t	you’d	desired	their	especially	toward
below	isn	so	you’ll	do	then	essentially	towards
between	isn’t	some	you’re	does	there	et al	typical
both	it	such	you’ve	each	thereby	etc	typically
but	it’s	t	your	embodiment	therefore	eventually	upon
by	its	than	yours	fig	thereof	excellent	via
can	itself	that	yourself	figs	thereto	finally	vice versa
couldn	just	that’ll	yourselves	for	these	furthermore	whatever
couldn’t	ll	the		from	they	good	whereas
d	m	their		further	this	hence	whereat
did	ma	theirs		generally	those	he/she	wherever
didn	me	them		had	thus	him/her	whether
didn’t	mightn	themselves		has	to	his/her	whose
do	mightn’t	then		have	use	ie	within
does	more	there		having	various	ii	without
doesn	most	these		herein	was	iii	yet
doesn’t	mustn	they		however	were	instead	
doing	mustn’t	this		if	what	later	
don	my	those		in	when	like	
don’t	myself	through		into	where	little	
down	needn	to		invention	whereby	many	
during	needn’t	too		is	wherein	may	
each	no	under		it	which	meanwhile	
few	nor	until		its	while	might	
for	not	up		means	who	moreover	
from	now	ve		not	will	much	
further	o	very		now	with	must	
had	of	was		of	would	never	
hadn	off	wasn		on		often	
