# Supplementary material for: Stopwords in technical language processing
Source: PLoS One. 2021 Aug 5;16(8):e0254937. doi: 10.1371/journal.pone.0254937 (PMC8341615; doi:10.1371/journal.pone.0254937)
Supplement: S1 Table — (PDF) [file pone.0254937.s001.pdf]

S1 Table. Top 30 terms for term-frequency, IDF, TFIDF and entropy.

	Term-Frequency (high to low)	IDF (low to high)	TFIDF (low to high)	Entropy (high to low)
1	include	include	include	include
2	first	method	one	method
3	method	one	method	one
4	device	device	comprise	device
5	second	first	form	comprise
6	one	second	least	first
7	form	comprise	device	system
8	apparatus	form	system	apparatus
9	system	system	apparatus	second
10	least	least	receive	form
11	may	apparatus	second	least
12	comprise	may	first	may
13	plurality	plurality	may	plurality
14	base	base	plurality	disclose
15	connect	receive	base	receive
16	receive	connect	connect	base
17	portion	disclose	within	connect
18	control	control	disclose	within
19	position	two	two	control
20	two	within	position	two
21	process	position	control	process
22	surface	process	generate	position
23	within	generate	determine	generate
24	generate	portion	extend	determine
25	configure	determine	portion	portion
26	determine	surface	make	make
27	signal	configure	surface	relate
28	end	extend	allow	extend
29	disclose	make	process	contain
30	data	relate	configure	surface
