# Supplementary material for: Stopwords in technical language processing
Source: PLoS One. 2021 Aug 5;16(8):e0254937. doi: 10.1371/journal.pone.0254937 (PMC8341615; doi:10.1371/journal.pone.0254937)
Supplement: S2 Table — * indicates that the term is also identified in the current study. + indicates that the term is a stopwords as defined in the current study. Rest of the terms are no longer considered as stopwords as defined in the current study. (PDF) [file pone.0254937.s002.pdf]

S2 Table. The stopwords identified in the previous study. * indicates that the term is also identified in the current study. + indicates that the term is a stopwords as defined in the current study. Rest of the terms are no longer considered as stopwords as defined in the current study.

able*	etc*	one another	therethrough*
above-mentioned+	eventually+	otherwise*	therewith*
already*	finally*	possibly	towards*
always*	furthermore*	rather*	typical+
and/or*	he/she+	remarkably+	via*
anything+	hence*	significantly+	vice versa+
anywhere+	him/her+	simply*	whatever+
better*	his/her+	sometimes+	whereat+
disclosure+	instead*	straight forward+	wherever+
easily*	may*	substantially	whether*
eg*	meanwhile+	therebetween*	whose*
either*	might+	therefor*	within*
elsewhere+	moreover+	therefrom*	without*
enough+	must*	therein*	wrt
especially*	often+	thereinto+	yet*
et al+	one	thereon*	
