# Supplementary material for: Stopwords in technical language processing
Source: PLoS One. 2021 Aug 5;16(8):e0254937. doi: 10.1371/journal.pone.0254937 (PMC8341615; doi:10.1371/journal.pone.0254937)
Supplement: S1 Nomenclature — (DOCX) [file pone.0254937.s003.docx]

**S1 Nomenclature**

| *δ* | discounting coefficient |
| --- | --- |
| *N* | total number of tokens in the patent database |
| *T_phrase_* | threshold variable used in phrasing method |
| *C* | corpus of patents |
| *t* | a single term in the corpus |
| *T* | use the “Tab” key to add more rows to this table |
| IDF | Inverse Document Frequency |
| LSTM | Long Short Term Memory |
| NLP | Natural Language Processing |
| NLTK | Natural Language Tool Kit |
| *TF* | Term Frequency |
| TFIDF | Term Frequency Inverse Document Frequency |
| USPTO | United States Patent and Trademark Office |
